# Phase contrast 4D flow in bicuspid aortic valves in a porcine model

**DOI:** 10.1186/1532-429X-17-S1-P416

**Published:** 2015-02-03

**Authors:** Matthias Grothoff, Dr Christian Etz, Bernhard Preim, Benjamin Köhler, Matthias Gutberlet

**Affiliations:** 1 Radiology, Leipzig Heart Center, Leipzig, Germany; 2 Cardiac Surgery, Leipzig Heart Center, Leipzig, Germany; 3 Computer Science, University Magdeburg, Magdeburg, Germany

## Background

Bicuspid aortic valves (BAV) are associated with aneurysms of the ascending aorta (AAA). It is unclear whether these aneurysms are caused by tissue alterations of the aortic wall or by alterations of blood flow in the ascending aorta due to the bicuspid valve morphology. In this study we analyzed the phase contrast 4D flow characteristics in normal tricuspid aortic valves (TAV) in a porcine model and compared them to the 4D flow patterns after different types of surgical bicuspidalization.

## Methods

Phase contrast 4D flow measurements of the thoracic aorta were performed in 3 mongrel swine (56 to 73 kg) using a spoiled gradient-echo based sequence prototype on a 3 Tesla MRI system (Verio, Siemens Healthcare, Erlangen, Germany) in supine position with a 16-channel cardiac phase-array coil. After the first MRI scan surgical bicuspidalization of the aortic valve was performed with a fusion of the right and left-coronary (R-L) leaflet in swine 1 and fusion of the right and the non-coronary (R-N) leaflet in swine 2. In swine 3 a congenital BAV of the R-L type was found intraoperatively. After surgery swine 1 and 2 underwent a second 4D flow scan. The 4D scans were analyzed for helical and vortical flow in the entire thoracic aorta and for eccentric flow at the level of the sinotubular junction.

## Results

**Figure 1:**
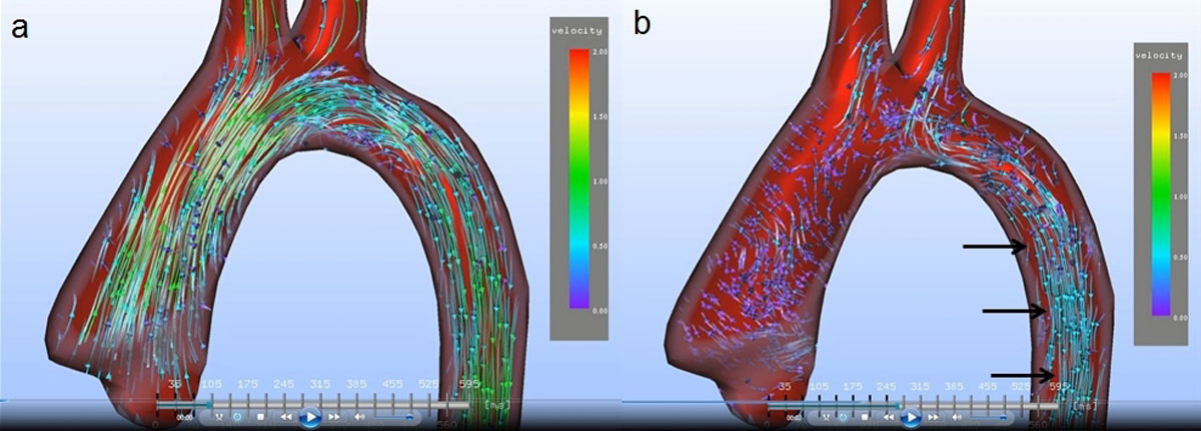
**Preoperative streamline analysis of pig 1 with a normal tricuspid aortic valve in systole (a) and early diastole (b).** In (a) laminar flow is shown in the ascending aorta, the aortic arch and the proximal descending aorta. No helical or vortical flow is present. In (b) laminar flow is still present in the proximal descending aorta in (black arrows).

Analysis of preoperative 4D flow characteristics in swine 1 and 2 revealed a predominantly laminar flow with high-velocity systolic streamlines parallel to the aortic wall and only discrete-right handed flow in the distal aortic arch and the descending aorta without helicicity (Fig. 1). No vortices and no eccentric flow at the level of the sinotubular junction could be detected. Swine 3 (congenital BAV) demonstrated a marked right-handed helical flow in peak systole.

**Figure 2:**
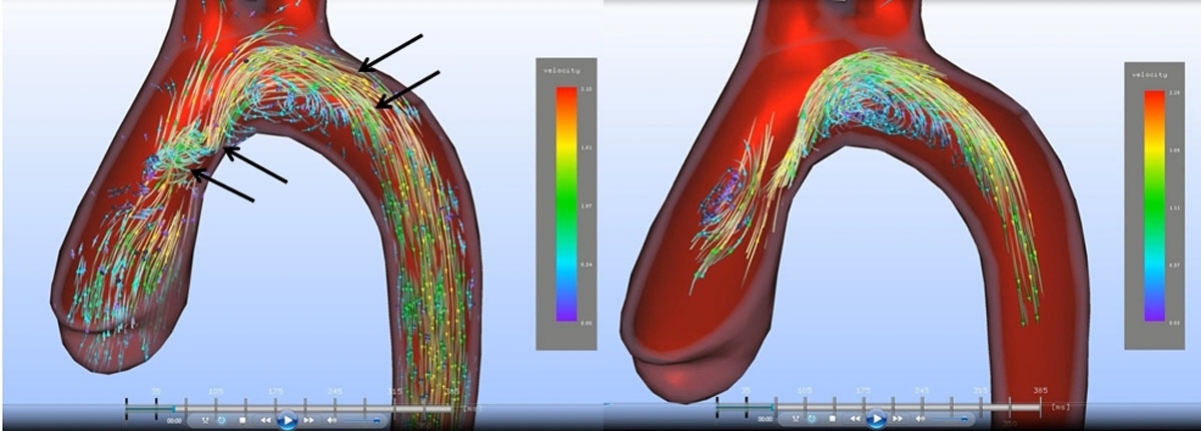
**Postoperative streamline analysis of pig 1 after bicuspidalization with right-left aortic leaflet fusion.** In (a) there is a right-handed helical flow in the ascending aorta and the distal aortic arch (arrows). In (b) vortices are extracted from the image data. A big vortex can be visualized in the aortic arch, a small vortex in the ascending aorta.

Postoperative analysis demonstrated substantial changes: In swine 1 (R-L) a right-handed helical flow was visible in the ascending aorta and the aortic arch with a corresponding eccentric flow with a jet towards the right-anterior quadrant at the level of the sinotubular junction. Extraction of vortices showed a pronounced vortex in the aortic arch and in the AA (Fig. 2). In swine 2 (R-N) an eccentric flow with a jet towards the left-posterior quadrant at the level of the sinotubular junction was visible and vortices could be detected in the AA.

## Conclusions

We could show that bicuspidalization of the aortic valve results in substantial changes of blood flow in the AA dependending on the type of leaflet fusion. This porcine model could be used to analyze the contribution of flow alterations in the development of AAA in BAV patients.

## Funding

None.

